# A Rare Cause of Cauda Equina Syndrome: Neuroendocrine Carcinoma of the Lung

**DOI:** 10.7759/cureus.69263

**Published:** 2024-09-12

**Authors:** Palmer H Ford, Eric Carbo, Andrew Rennie, Richard Virgilio

**Affiliations:** 1 Department of Internal Medicine, Brookwood Baptist Health, Birmingham, USA; 2 Department of Internal Medicine, Edward Via College of Osteopathic Medicine, Auburn, USA; 3 Department of Internal Medicine, Grandview Medical Center, Birmingham, USA; 4 Department of Clinical Affairs, Edward Via College of Osteopathic Medicine, Auburn, USA

**Keywords:** small cell lung cancer (sclc), neuroendocrine carcinoma (nec), stage iv cancer, pulmonology, intramedullary spinal cord metastasis, lung cancer, chemotherapy, leptomeningeal carcinoma, cauda equina syndrome (ces), large cell neuroendocrine

## Abstract

Cauda equina syndrome (CES) is a rare condition describing the constellation of symptoms resulting from the compression of the cauda equina. Metastatic lesions are a common cause of CES, with lung lesions often implicated as the primary source. A particularly rare cause of CES is leptomeningeal metastasis (LM) from primary solid tumors. In this case, a 63-year-old male presented with urinary and fecal retention, as well as altered sensation in the genitalia. The clinical diagnosis of CES was based on the constellation of symptoms. Computed tomography (CT) imaging demonstrated a metastatic lesion in the S2 and S3 sacral vertebral bodies, with extension into the right piriformis muscle. Magnetic resonance imaging (MRI) revealed an intramedullary lesion at L2 and leptomeningeal enhancement, indicative of metastasis. Further imaging identified a primary lesion in the right lower lobe of the lung, with additional metastases to the brain and liver. A pathological diagnosis of metastatic neuroendocrine carcinoma (NEC) was confirmed following a supraclavicular lymph node biopsy. The patient received steroid therapy, chemotherapy, and radiation to the pelvis. This case provides an important perspective on CES evaluation due to the scarcity of literature highlighting spinal metastases as the primary presentation in patients with NEC of the lung. The clinical diagnosis of CES should raise suspicion for metastasis and warrant further investigation.

## Introduction

The cauda equina is the collection of nerve roots at the end of the spinal cord. Dysfunction of the cauda equina can cause motor and sensory dysfunction at the level of the lesion. Cauda equina syndrome (CES) is most commonly due to compression, infection, or birth abnormalities. It can be a highly distressing presenting set of symptoms of a primary lung malignancy that has metastasized to the spine. Symptoms of CES commonly include urinary retention, fecal or urinary incontinence, saddle anesthesia, weakness in any affected nerve root distribution, pain in the back, and sexual dysfunction [1]. According to the literature, it is estimated that 45% of CES cases are due to compression by a herniated lumbar intervertebral disc, while malignancy is estimated to be implicated in 1-8% of cases [2].

Large cell neuroendocrine carcinoma (LCNEC) of the lung is a highly aggressive tumor that typically arises in patients with an extensive smoking history and acts similarly to small cell lung carcinoma (SCLC). The incidence of LCNEC in resected lung cancers is estimated to be between 2.1% and 3.5% [3]. SCLC is a high-grade, poorly differentiated neuroendocrine tumor that typically presents as a primary pulmonary neoplasm near the bronchial region. SCLC is more common than LCNEC and accounts for 10-15% of all lung cancers [4,5]. LCNEC has a unique histological presence, which is the basis for its diagnosis and is related to other neuroendocrine tumors. Among these histological characteristics are positive staining for markers such as chromogranin A, synaptophysin, or CD56 [6]. LCNECs can arise in many areas throughout the body, such as the gastrointestinal tract, brain, and lung, with the lung being the most common location [7].

Bone metastases are common in breast, lung, prostate, kidney, and hematopoietic tumors, with a specific predilection for metastasis to the spine. Up to 70% of documented cancer diagnoses can have secondary spinal lesions [8]. Leptomeningeal metastasis (LM) is a devastating complication of metastatic disease, which occurs when neoplastic cells invade the meningeal space. LM can be found in less than 5% of patients with malignant solid tumors; however, literature shows an incidence of approximately 20% or more on postmortem examination of patients with solid tumors [9]. Leptomeningeal carcinomatosis is a serious complication, with a median survival of 1.3 months from the time of diagnosis, without appropriate therapy [10].

## Case presentation

A 63-year-old male with a 40-pack-year smoking history presented to the Emergency Department with a two-week history of urinary and fecal retention, accompanied by an altered sensation of the genital region. Past medical history noted hypertension, hyperlipidemia, and alcohol use disorder. He was initially seen a month prior to this presentation at an outside hospital for urinary retention, with normal sensation in the genital area, and was treated with placement of a Foley catheter, which drained 3 L of urine, and tamsulosin. He was discharged from the Emergency Department with complete resolution of symptoms and no further workup. On physical exam, he had mild motor weakness with dorsiflexion and extension of the right foot (4/5 strength), indicating mild foot drop. The sensation of the lower extremities was normal bilaterally. He had zero sensation of the penis and genital area, along with decreased rectal tone. Clinically, he met the criteria for the diagnosis of CES. He denied any hemoptysis, shortness of breath, chest pain, cough, fevers, night sweats, weight loss, or nausea.

**Figure 1:**
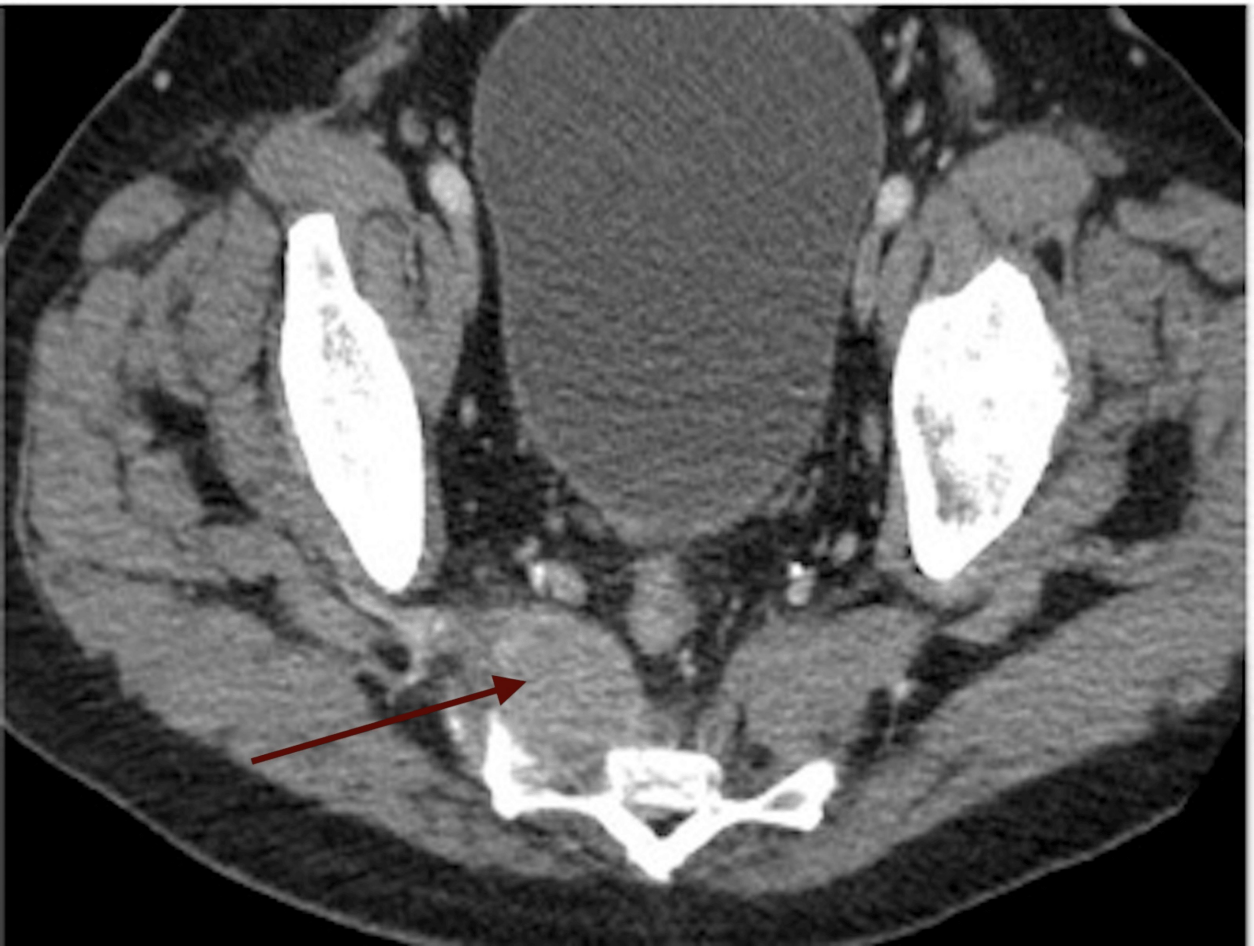
CT image of the lesion at the level of S2-S3 sacral body with malignant extension into the piriformis muscle The red arrow indicates a metastasized tumor within the piriformis muscle. CT: Computed tomography

Imaging was emergently obtained by way of computed tomography (CT). CT demonstrated a metastatic lesion of the S2-S3 sacral vertebral bodies, with extension into the right piriformis muscle (Figure 1).

**Figure 2:**
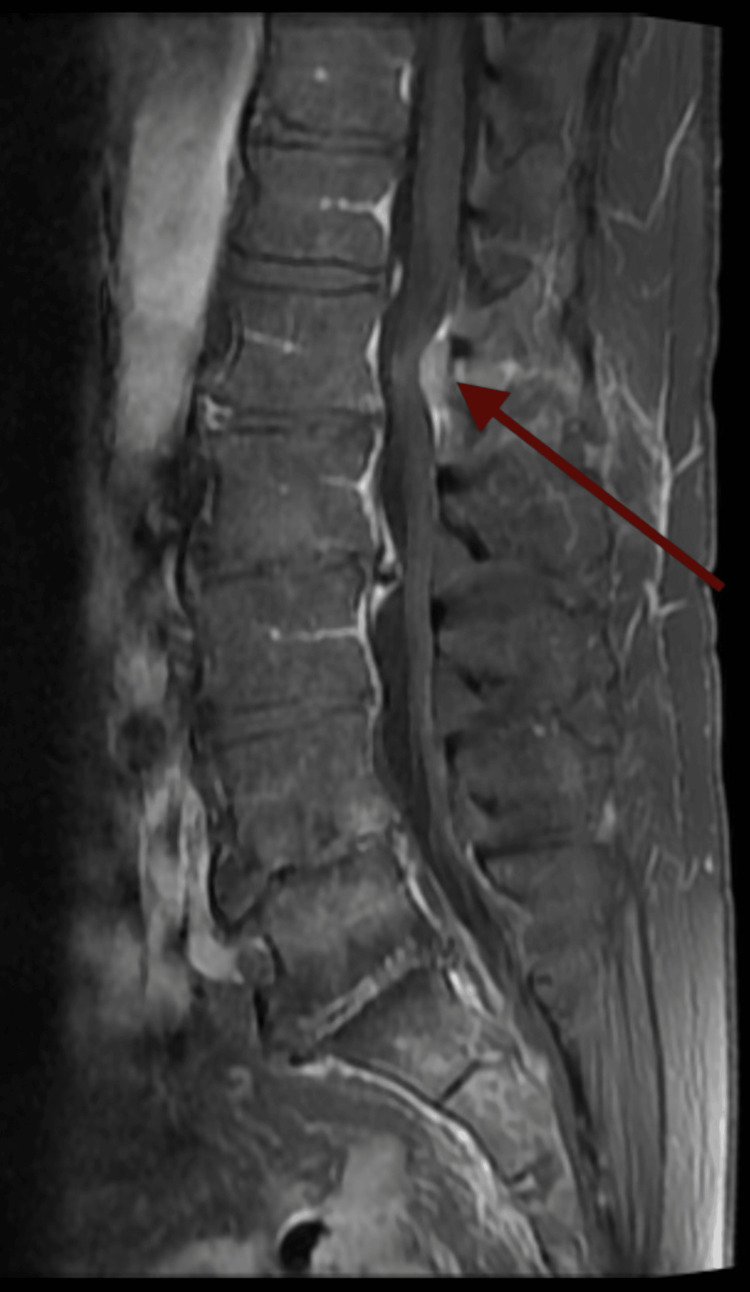
MRI demonstrating intramedullary spinal cord lesion at the level of L2 The red arrow depicts a metastatic lesion infiltrating the intramedullary space. MRI: Magnetic resonance imaging

**Figure 3:**
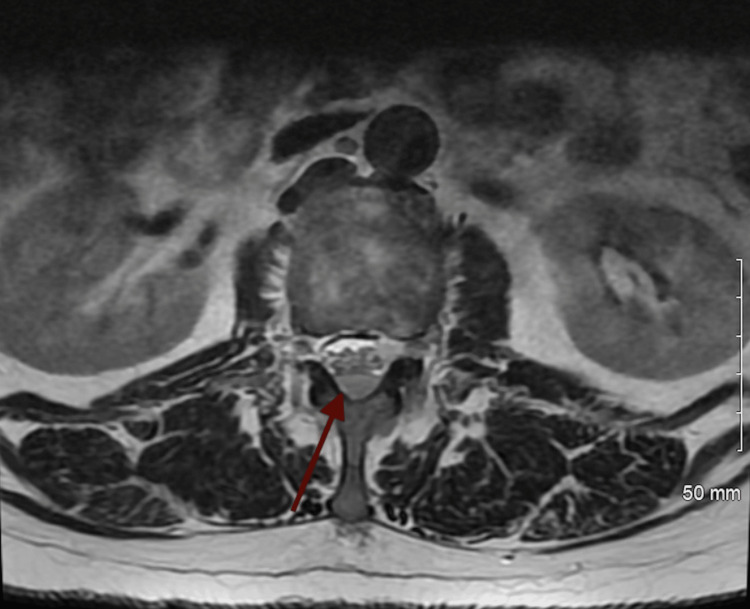
MRI imaging showing mild enhancement of the cauda equina nerve roots, suspicious for carcinomatosis The red arrow depicts the area of enhancement, shown as white discoloration of the cauda equina, demonstrating malignant carcinomatosis. MRI: Magnetic resonance imaging

Due to this finding, magnetic resonance imaging (MRI) of the abdomen and pelvis was then obtained. MRI revealed mild enhancement of the cauda equina nerve roots, raising suspicion for meningeal carcinomatosis (Figure 2), as well as an intramedullary lesion at the level of L2 (Figure 3).

**Figure 4:**
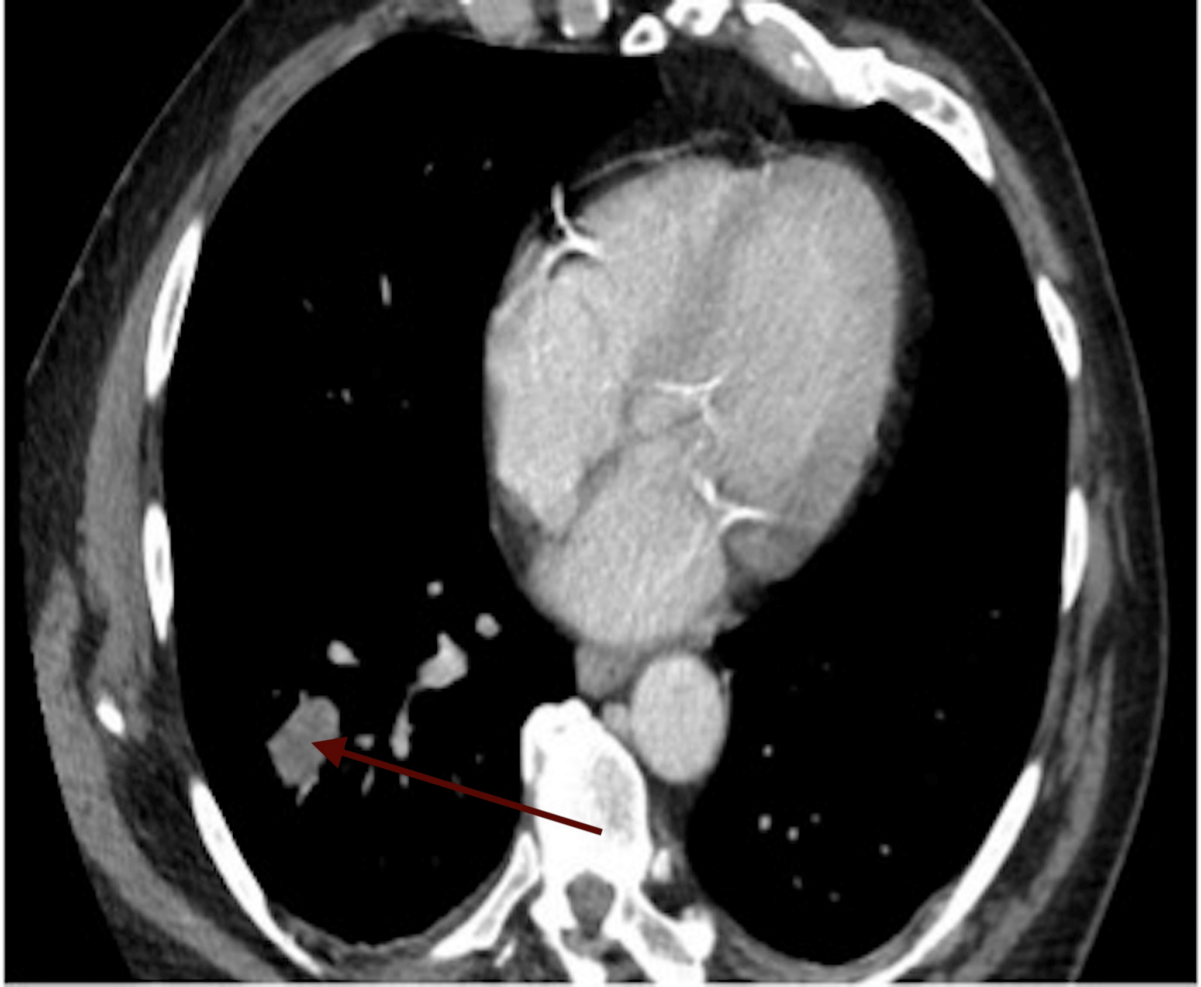
CT image of the primary lesion incidentally found on additional imaging The red arrow depicts a malignant lesion of the right lung. CT: Computed tomography

**Figure 5:**
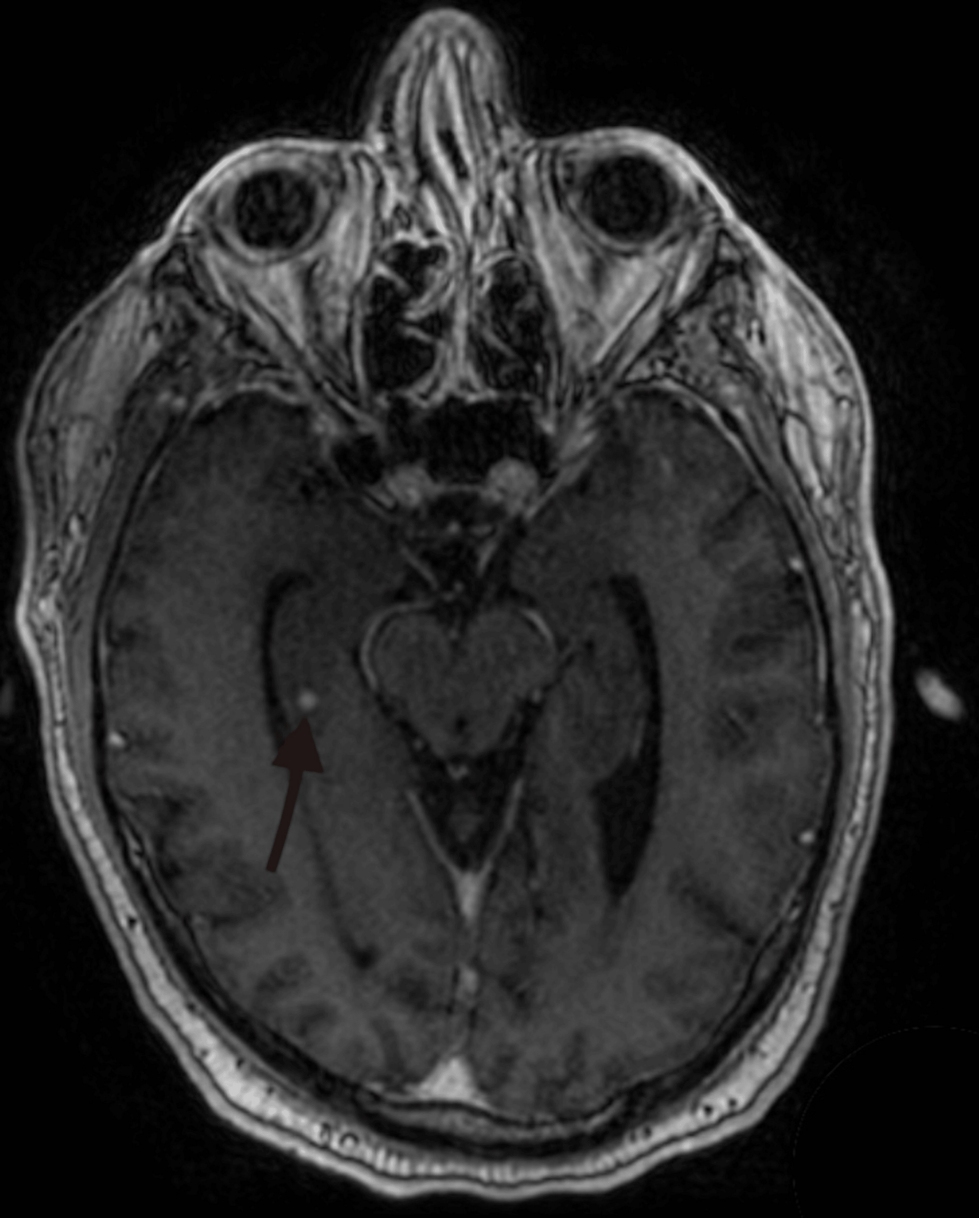
MRI image displaying metastatic lesion in the brain The red arrow depicts a lesion found within the right temporal cortex. MRI: Magnetic resonance imaging

Additional imaging was then required due to these findings, which included chest CT and brain MRI. This further imaging revealed a 2.8 cm solid primary lesion in the right lower lobe of his lung (Figure 4), and an MRI of his brain demonstrated a 3 mm solitary focus within the right temporal cortex (Figure 5), suspicious of metastatic disease.

**Figure 6:**
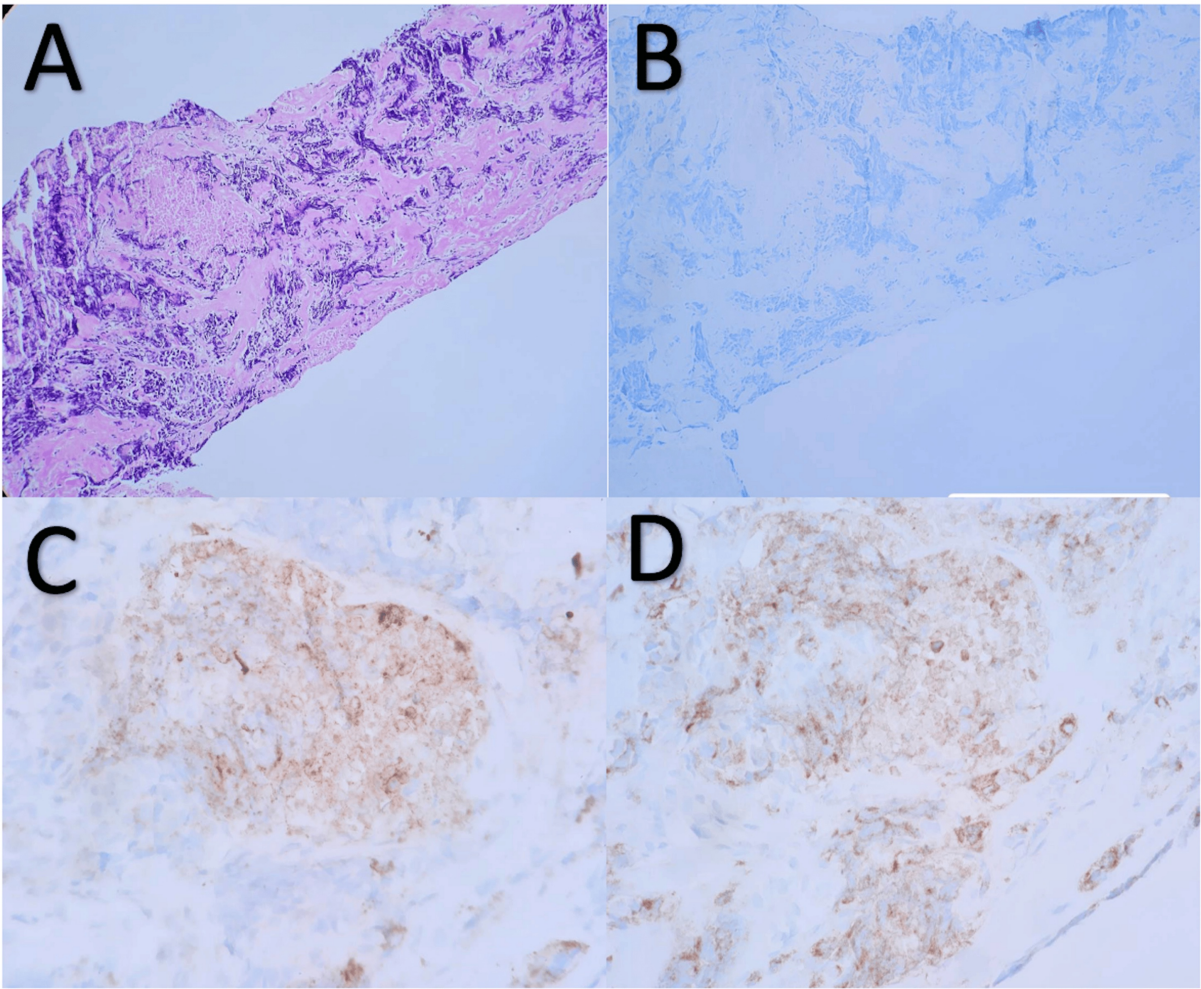
Pathological stains used to establish a diagnosis A) H&E stain at 10x; B) Chromogranin A, resulted negative; C) CD56, resulted positive; D) Synaptophysin, resulted positive

A tissue sample, measuring 1.3 cm in length and 0.1 cm in diameter, was retrieved via supraclavicular lymph node biopsy, and a pathological diagnosis of metastatic neuroendocrine carcinoma (NEC) was made. Further staining was unable to distinguish between LCNEC and SCLC (Figure 6). After staging, the malignant NEC was labeled Stage IVB, T1c, N3, and M1c.

During this admission, he was treated with high-dose corticosteroids in the hospital, with hopes of alleviating symptomatology, as well as placement of a Foley catheter to drain urine and alleviate retention. Upon consultation with oncology, he was immediately started on chemotherapy in the inpatient setting, with plans for outpatient palliative radiation to the pelvis following his discharge from the hospital. His inpatient chemotherapy regimen included carboplatin and etoposide, with a combination of high-dose steroids and Foley placement. The patient had an 18-day stay in the hospital, complicated by an illness with respiratory syncytial virus (RSV). The patient was able to be discharged home after 18 days, with an indwelling catheter and opioids for pain management. Symptomatology mildly improved before discharge, and radiation therapy was begun in an outpatient setting, as well as continuation of chemotherapy. This combination of therapies has led to an increased quality of life, as stated by the patient. 

## Discussion

NEC of the lung is not commonly seen and rarely presents with metastasis to the spine. This patient had no findings or symptoms prior to admission that suggested metastatic disease, other than his prior extensive smoking history. As noted in a systematic review of 9,876 cases of lung cancer, not a single case had presenting symptomatology related to CES as the sole symptom at presentation [11]. This presentation highlights another potential initial symptom that clinicians can consider when forming a differential diagnosis. Lung cancer five-year survival rates have increased by 22-26.6% from 2016 to 2019, underscoring the advancement in treatment modalities. Due to this increased survival, the risk of spinal metastasis rises, which in turn increases the risk of CES [12]. In this case, the patient experienced two weeks of CES symptoms that progressively worsened. In aggressive lung carcinomas such as LCNEC and SCLC, CES may become a more prevalent primary presenting syndrome. Therefore, physicians should be more aware of potential aggressive metastasis to the spine in patients with extensive smoking history. Early involvement of neurological surgery services and additional imaging modalities could potentially improve the five-year survival probability.

Of the sites of metastases in this patient, the most devastating is the seeding of the leptomeninges. In a previous literature review on lung cancer spread causing CES, only 131 cases were identified, with LMs carrying the worst prognosis: six months with normal cerebrospinal fluid movement and four months with interrupted cerebrospinal fluid flow [13]. Clinically diagnosed in only 5% of solid malignant tumor cancers and found in 20% of post-mortem examinations, LM in high-grade carcinomas can be safely assumed to be grossly underdiagnosed [9]. According to Liu et al., there is no described gold standard for diagnosing LM and carcinomatosis; however, gadolinium-enhanced MRI will show abnormal enhancement of the leptomeninges, making it an important index for radiologists to consider when reviewing studies in patients with CES [14]. Our patient had a metastatic lesion of the S2-S3 sacral vertebral bodies with extension into the right piriformis muscle, as well as enhancement of the cauda equina nerve roots. Other studies have highlighted the importance of 18F-fluorodeoxyglucose positron emission tomography-computed tomography (FDG PET/CT) as a primary modality to detect LM, making this imaging modality another strong option after initial MRI detection [3]. This patient did not receive a PET scan during his hospital admission and was deemed to have it considered on an outpatient basis, as an MRI was sufficient to begin therapy. Although not proven to alter the survival course, assessing LMs could potentially help tailor treatment courses in patients with CES to improve their quality of life.

Due to the lack of literature surrounding this diagnosis, this case report aims to add to the medical literature further examples of patient presentation, potential management, and patient outcomes. While exact management is dependent on each unique presentation and clinical picture, the first study published on this topic, which involved surgical decompression, resulted in poor functional outcomes [8]. Treatment modalities and outcomes are still to be researched to establish the overall best course of action to improve quality of life and increase survival. A recent study found that spine LMs from primary non-central nervous system (CNS) lesions had a worse prognosis than intracranial LMs from non-primary CNS lesions. This study also showed, in a systematic review of 72 cases of LMs, that locoregional radiotherapy, chemotherapy, and surgical decompression were performed for palliation in most cases, with outcomes remaining dismal and prognoses discouraging [15]. Previous studies have now demonstrated better survival advantages with non-operative treatment approaches in this patient population, and this is the approach taken with our specific case [16].

## Conclusions

Newly emerging CES could be a sign of metastasis to the leptomeninges of a malignant carcinoma, such as NECs of the lung, presenting a challenging condition. This case of CES secondary to metastatic lung neuroendocrine tumors highlights the critical need for prompt diagnosis and a multidisciplinary approach to treatment. Palliative management with non-operative strategies illustrates the potential benefits of such approaches in improving patient quality of life. Recognizing rare metastatic causes of CES in patients with a history of malignancy can significantly enhance diagnostic accuracy and therapeutic effectiveness in medical practice.
